# PERSONALITY MODERATES INTERVENTION EFFECTS ON COGNITIVE FUNCTION: A 6-WEEK CONVERSATION-BASED INTERVENTION

**DOI:** 10.1093/geroni/igz038.828

**Published:** 2019-11-08

**Authors:** Eric S Cerino, Karen Hooker, Elena Goodrich, Hiroko H Dodge

**Affiliations:** 1 Oregon State University, Corvallis, United States; 2 Oregon state University, Corvallis, Oregon, United States; 3 Layton Aging and Alzheimer’s Disease Center, Oregon Health and Science University, Portland, Oregon, United States; 4 Department of Neurology, Layton Aging and Alzheimer’s Disease Center, Oregon Health and Science University, Portland, Oregon, United States

## Abstract

Conversation-based interventions have positive effects on cognitive health, though determining who benefits most is still unclear, and individuals’ personality may play a role. We utilized data from a 6-week randomized controlled trial to determine if conversation-based intervention effects were moderated by personality traits in 83 older adults (Mean age = 80.51 years, 49 cognitively intact, 34 with mild cognitive impairment). The intervention group participated in daily 30-minute face-to-face semi-structured conversations with trained interviewers for six weeks. Baseline psychosocial questionnaires and a neuropsychological battery were completed. Intervention group participants with high agreeableness, conscientiousness, and extraversion exhibited significantly more improvement in language-based executive function tasks compared to a control group (ps<.05). An opposite pattern for delayed recall memory and working memory tasks emerged among highly extraverted participants (ps<.05). Findings suggest the adaptive role of personality traits in conversation-based cognitive interventions and offer evidence for personalized approaches to cognitive health in late life.

